# Influence of cultivation duration on microbial taxa aggregation in *Panax ginseng* soils across ecological niches

**DOI:** 10.3389/fmicb.2023.1284191

**Published:** 2024-01-10

**Authors:** Zhenting Shi, Meling Yang, Kexin Li, Li Yang, Limin Yang

**Affiliations:** Cultivation Base of State Key Laboratory for Ecological Restoration and Ecosystem Management, College of Traditional Chinese Medicine, Jilin Agricultural University, Changchun, China

**Keywords:** *Panax ginseng*, ecological niche, bacteria, fungi, microbiome, crop succession disorder

## Abstract

**Introduction:**

Microbial communities are crucial for plant health and productivity. However, the influence of cultivation age on the ecological processes in assembling plant microbiomes at various ecological niches remains unclear.

**Methods:**

We selected 12 samples from ginseng farmlands with different cultivation years (N4: 4 years old, N6: 6 years old). We used soil physicochemical properties, enzyme activities, and high-throughput sequencing (16S rDNA and ITS) to examine the rhizoplane (RP), rhizosphere (RS), and bulk soil (BS).

**Results:**

Our results indicated that cultivation years significantly affect the soil microbiome’s diversity and community composition across different ecological niches. The BS microbiome experienced the largest effect, while the RS experienced the smallest. N6 showed a greater impact than N4. This effect was more pronounced on the fungal communities than the bacterial communities of various ecological niches and can be closely related to the soil’s physicochemical properties. In N4 soils, we observed an upward trend in both the number of ASVs (amplicon sequence variations) and the diversity of soil microbial taxa across various ecological niches. In N4RP, the bacteria *Sphingomonas*, known for degrading toxic soil compounds, was present. All ecological niches in N4 showed significant enrichment of *Tetracladium* fungi, positively associated with crop yield (N4RP at 6.41%, N4RS at 11.31%, and N4BS at 3.45%). In N6 soils, we noted a stark decline in fungal diversity within the BS, with a 57.5% reduction in ASVs. Moreover, *Sphingomonas* was abundantly present in N6RS and N6BS soils. The relative abundance of the pathogen-inhibiting fungus *Exophiala* in N6RP and N6RS reached 34.18% and 13.71%, respectively, marking increases of 4.9-fold and 7.7-fold. Additionally, another pathogeninhibiting fungus, *Humicola*, showed significant enrichment in N6BS, with a 7.5-fold increase. The phenolic acid-producing fungus *Pseudogymnoascus* in N6RP, N6RS, and N6BS showed increases of 2.41-fold, 2.55-fold, and 4.32-fold, respectively. We hypothesize that functional genes related to the metabolism of terpenoids and polyketides, as well as signaling molecules and interactions, regulate soil microbial taxa in ginseng from different cultivation years.

**Discussion:**

In conclusion, our study enhances understanding of plant-microbe interactions and aids the sustainable development of medicinal plants, particularly by addressing ginseng succession disorder.

## 1 Introduction

Microorganisms have co-evolved with their hosts for more than 400 million years (Hu et al., 2020; Santoyo, 2022). They play a key role in maintaining plant health and productivity (Hassani et al., 2018; Chica et al., 2019; Xiong et al., 2021a). Soil, environment, and host factors (such as genotype, ecological niche, etc.) influence microbial taxa. Studies have shown that the ecological niche plays a critical role in special conditions (Gao et al., 2021; Xiong et al., 2021a,b). Researchers propose a multi-step selection model for inter-root microbial communities. This model suggests sequential selection in bulk soil (BS), rhizosphere (RS), and root to form specific plant RS microbial communities (Tian et al., 2020; Wang et al., 2020). In this process, the host selection effect intensifies from BS to rhizoplane (RP), while bacterial community diversity and network complexity decrease (Xiong et al., 2021b). The importance of ecological niches at varying root-soil distances is clear. However, the impact of cultivation years on microbial taxa patterns is less explored. Understanding this process is vital for the temporal dynamics of plant microbiome assembly and for developing sustainable production systems (Busby et al., 2017; Singh et al., 2020; Haskett et al., 2021; Xiong et al., 2021a).

Ginseng, the “king of herbs,” is a perennial plant (*Panax ginseng* C. A. Mey.) and a popular traditional Chinese medicine. Ginseng is harvested after 4–5 years of cultivation. However, it faces a severe succession disorder. Land once planted with ginseng requires over 30 years of restoration before replanting, or it risks diseases and yield loss (Yang et al., 2004; Shi Z. T. et al., 2022). Meanwhile, replant methods have led to deforestation and ecological damage, highlighting a conflict between ecology and the economy (Yang et al., 2000). The succession problem affects both ginseng industry sustainability and forest conservation (Yang et al., 2004). Therefore, ecological principles must be applied to achieve sustainable agricultural systems. Studies have linked succession disorder to soil microbiota changes (Wei et al., 2020; Gao et al., 2021), pathogen increases (Weidenhamer, 1996; Wei et al., 2022), soil property deterioration (Xiao et al., 2016; Vives-Peris et al., 2018; Liu et al., 2021), and ginseng secretions’ effects (Sun et al., 2008). We hypothesize that ginseng soil microbial communities differ with cultivation years and ecological niches of the root. This difference could contribute to the succession disorder.

However, most previous studies focused on ginseng BS or RS microecology, ignoring the RP (Wang et al., 2019; Tong et al., 2021; He et al., 2022; Jin et al., 2022). Currently, there are lots of studies comparing the microbial communities in the BS, RS, and RP (Pérez-Izquierdo et al., 2019; Yu et al., 2021). However, the knowledge about bacterial and fungal communities changing in different plant stages and the key species involved in maintaining the health and yield of ginseng is still unknown. This lack of information seriously limits RS microecology progress and sustainable resource development (Yang et al., 2000). Therefore, we investigated microbial community changes in ginseng soils at various ecological niches and cultivation years using 16S rDNA and ITS analyses. This will help understand plant-microbe interactions and provide insights for managing succession disorders in medicinal plants.

## 2 Materials and methods

### 2.1 Field experiments and sample collection

Samples were collected from the ginseng main production area located in Fusong County, Jilin Province, China (N42°22′36.74″E127°06′37.59″). The soil type was black loam, and the soil background was corn farmland that is older than 20 years, with traditional planting and fertilization practices from the main production area. The region has a temperate continental monsoon climate with an average annual rainfall of 800 mm. On September 16, 2021, we collected samples of 4 years-old (3 years-old seedlings transplanted for 1 year; N4) and 6 years-old (3 years-old seedlings transplanted for 3 years; N6) ginseng, respectively. We found a significant difference in the yield of ginseng between the N4 and N6 stages of plants (Supplementary Table S1), and the continuous cropping obstacle gradually increased with the number of years of transplanting. A randomized grouping design was used, with six biological replicates of samples from each cultivation year, and a total of 12 plots of 20 m^2^ each. The collection followed a five-point sampling method and ensured that over 20 healthy ginsengs were selected for each replicate, amounting to over 250 ginsengs and soil samples.

### 2.2 Sample handling

All used utensils (pry, sample bag, brushes, cryopreservation tube, etc.) were sterilized. We first removed the top layer of soil, about 10 cm from the ginseng (at a depth of about 20 cm), digging out the ginseng intact and gently shaking the harvested roots. The shaken-off soil was defined as the BS. BS samples were sieved through a 20-mesh sieve and all samples were numbered and placed in a sampling box containing ice packs. They were transported back to the laboratory on the same day for further processing. We used a brush to remove the soil within 2 mm immediately adjacent to the ginseng roots, a portion referred to as the RS soil (Huang et al., 2022). After removing the RS soil, each sample was rinsed with ultrasonic shock (30 s, 50–60 Hz) using PBS buffer (phosphate buffer saline). The rinsate was subjected to high-speed centrifugation (6,000 × g, 4°C) for 20 min to obtain the RP soil (Edwards et al., 2015; Qin et al., 2018). Samples from both years six replicates for RS soil (samples named N4RS and N6RS) and five replicates for BS (samples named N4BSand N6BS) and RP (samples named N4RP and N6RP) were divided into two parts, totaling 64 sets of samples. One part was used for 16S rDNA and ITS detection and stored at −80°C for DNA extraction. The other part of the RS and BS samples, a portion of which was used for water content determination, was air-dried for physicochemical indices and enzyme activity analyses. The fresh weight of ginseng was determined and the results are shown in Supplementary Table S1.

### 2.3 Soil physical and chemical properties and enzyme activity tests

We tested the soil’s physical and chemical properties by estimating the soil water content (SWC) using the drying method, pH using the potentiometric method, electrical conductivity (EC) using the electrode method, soil organic matter (SOM) using the direct heating method, total nitrogen (TN) using the automatic nitrogen fixation method, total phosphorus (TP) using the sodium hydroxide alkali-soluble molybdenum antimony colorimetric method, total potassium (TK) using the sodium hydroxide melting method, available nitrogen (AN) using the alkali dissolution diffusion method, and available phosphorus (AP) using the UV/visible spectrophotometer method (Bao, 2000). The enzyme activities were determined as follows: soil cellulase (S-CL) and soil sucrase (S-SC) using the 3,5-dinitrosalicylic acid colorimetric method, soil β-glucosidase (S-β-GC) using the nitrophenol colorimetric method, acid protease (S-AcPr) using the Finsler method, soil urease (S-UE) using the indophenol blue colorimetric method, soil acid phosphatase (S-ACP) using the colorimetric phenyl disodium phosphate method, soil catalase (S-CAT) using the ultraviolet absorption method, and soil dehydrogenase (S-DHA) using the 2,3,5-triphenyl tetrazolium chloride (TTC) colorimetric method; all kits were supplied by Solarbio (Beijing, China).

### 2.4 DNA extraction, PCR amplification, and high-throughput sequencing

We used the kit extraction method to extract DNA from each sample, with a blank control using non-nucleated water. All extracted total DNA was eluted using 50 μL of elution buffer and stored at −80°C and set aside for amplification with PCR. For the V3–V4 region of the 16S rDNA (Logue et al., 2016), we selected the following primers: 341F (5′-CCTACGGGGNGGCWGCAG-3′) 805R (5′-GACTACHVGGGGTATCTAATCC-3′). For ITS, ITS2 (Yao et al., 2019) primers were used: ITS1FI2 (5′-GTGARTCATCGAATCTTTG-3′) ITS2 (5′-TCCTCCGCTTATTGATATATGC-3′). PCR amplification was performed in a 25 μL reaction system containing 25 ng of template DNA, 12.5 μL of PCR Premix, 2.5 μL of each primer, and a regulated volume of PCR grade water. PCR conditions for amplification of prokaryotic 16S fragments included initial denaturation at 98°C for 30 s, denaturation at 98°C for 10 s, annealing at 54°C for 30 s, extension at 72°C for 45 s for a total of 32 cycles, and a final extension at 72°C for 10 min. The PCR products were confirmed by electrophoresis on a 2% agarose gel. The PCR products were confirmed by 2% agarose gel electrophoresis. Ultrapure water was used as a negative control instead of sample solution to ensure the accuracy of the PCR results, and the PCR products were purified using AMPure XT beads (Beckman Coulter Genomics, Danvers, MA, United States) and subsequently quantified using Qubit (Invitrogen, United States). Finally, all samples were sequenced on the Illumina NovaSeq platform according to the manufacturer’s recommendations (the sequencing service was provided by LC-Bio, Hangzhou, China).

### 2.5 Data analysis

Samples were sequenced using the Illumina NovaSeq platform (LC-Bio., China). Corresponding paired-end reads were grouped into samples by unique barcodes, and barcodes and primer sequences were removed. Paired-end reads were merged using FLASH. For raw data, we used fqtrim (v0.94) for quality filtering to ensure high-quality reads. Chimeric sequences were filtered using Vsearch software (v2.3.4). Subsequently, the relative abundance of bacteria and fungi for each sample was normalized against feature abundance using the SILVA (release 138) classifier, the RDP database, and the UNITE database.

Based on the ASV (amplicon sequence variation) feature sequence and abundance tables, we performed alpha and beta diversity analyses. To assess microbial richness and diversity, the Chao1 and Shannon diversity indices were used. Differences between groups were analyzed using the Kruskal–Wallis test. The Bray–Curtis differences in the community matrix of ASVs were presented in two dimensions by NMDS (nonmetric multidimensional scaling). These were analyzed in conjunction with PERMANVOVA. This analysis focused on the reasons for differences in the samples, which were tested for significance using the permutation test. Stacked plots categorized the relative abundance of TOP30 species, presenting the relative abundance of each sample in a different form. LEfSe analysis of variance was conducted to find out ecological niche-specific biomarkers. The relationship between soil chemical properties, enzyme activities, and relative abundance of microorganisms corresponding to different cultivation years in the RS and BS was analyzed using the Spearman correlation coefficient and is presented as clustered heatmaps. Functional annotation was performed based on PICRUSt2 functional predictions using the KEGG database. The data images, including ASV petal plots, alpha diversity and violin plots, stacked plots, LEfSe variance analysis, relative abundance clustering heatmaps, and STAMP variance analysis plots, were analyzed using the “plotrix,” “ggplot2 (3.2.0), vegan, nsegata-lefse, and corrplot packages in R-3.4.4, respectively. All other graphs were plotted using the R package (v3.5.2). All data have been uploaded to NCBI under the registration numbers PRJNA1004743 for bacteria and PRJNA1004746 for fungi.

## 3 Results

### 3.1 Changes in soil physicochemical properties

We determined the soil physicochemical properties of the N4RS, N4BS, N6RS, and N6BS samples. The results showed that pH, SOM, TN, TP, AN, and TK in the RS and BS showed a decreasing trend with the increasing years of cultivation (i.e., N4RS > N6RS, N4BS > N6BS). Among them, pH was significantly higher in N4RS than in other groups. EC, TN, and AN exhibited significant differences among the four groups of samples. In all groups, TN and AN decreased significantly with increasing years of cultivation (N4RS > N6RS, N4BS > N6BS). In contrast, EC showed the opposite trend and increased significantly with increasing years of cultivation (N4RS < N6RS, N4BS < N6BS). In all groups, EC, TN, and AN were significantly lower in BS than in the RS (N4RS < N4BS, N6RS < N6BS), except TN in N4RS, which was higher than in N4BS. SOM decreased significantly with increasing years of cultivation (N4 > N6) but without significant differences between N4RS and N4BS or N6RS and N6BS samples. Among all groups, SOM, TP, OP, TN, and AN were lowest in N6RS, while OP, TN, and AN showed significant change. TK was significantly lower in N6BS than in other groups (Table 1).

**Table 1:** Physicochemical properties of rhizosphere and bulk soil across groups.

	Samples	pH	EC (ms/cm)	SOM (g/kg)	T N (g/kg)	AN (mg/kg)	TP (g/kg)	AP (mg/kg)	TK (g/kg)
N4	Rhizosphere	6.381 ± 0.2190a	1.15 ± 0.0240d	51.96 ± 11.99a	9.182 ± 0.01778a	145.8 ± 0.4041b	0.7551 ± 0.0302a	14.91 ± 1.938a	24.67 ± 2.363a
	Bulk soil	5.807 ± 0.2265b	1.918 ± 0.0106b	51.58 ± 5.467a	8.589 ± 0.0056b	152.6 ± 0a	1.29 ± 0.0302a	14.61 ± 0.105a	23.69 ± 1.005a
N6	Rhizosphere	5.781 ± 0.209b	1.783 ± 0.0101c	24.46 ± 1.505b	5.372 ± 0.0023d	82.6 ± 14d	0.7302 ± 0.0877a	6.415 ± 0.2741b	22.54 ± 0.1897ab
	Bulk soil	5.756 ± 0.0925b	2.463 ± 0.0208a	27.12 ± 2.154b	5.665 ± 0.02728c	100.1 ± 3.897c	1.241 ± 0.4522a	12.7 ± 0.3771a	19.78 ± 2.621b

Accompanied by the same letter following by Duncan’s new multiple range test are not statistically different (*p* = 0.05 a, b, and ab are significant markers, different letters indicate significant differences, as long as one letter is the same, it is not significant), N4 (4 years-old farmland ginseng), N6 (6 years-old farmland ginseng), electrical conductance (EC), soil organic matter (SOM), total nitrogen (TN), total phosphorus (TP), total potassium (TK), available nitrogen (AN), and available phosphorus (OP).

### 3.2 Analysis of soil microbial composition

In this study, 2,410,802 bacterial and 2,399,495 fungal validated sequences were obtained from 64 samples. These were categorized into 21,587 bacterial and 6,346 fungal ASVs.

**Figure 1:**
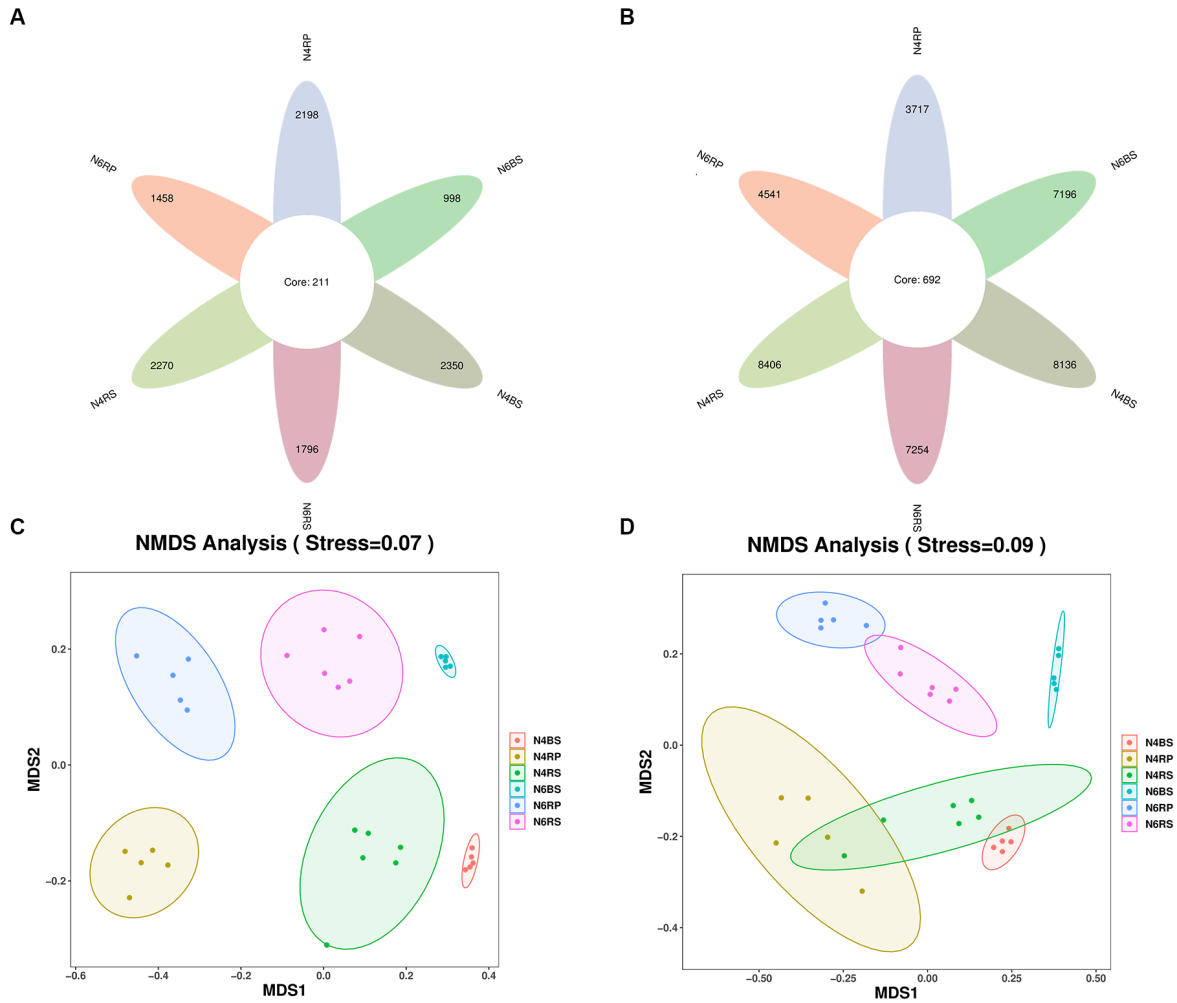
Changes in the number of ASVs and beta-diversity in all groups of soil samples. The petal diagram **(A)** shows bacteria and **(B)** shows fungi, NMDS showed the structure of the bacteria **(C)** and fungal **(D)** community among different planting years and ecological niches of ginseng root soil.

We found significant differences in the number of bacterial ASVs between ecological niches. There were lower ASVs in the RP than in the RS. For example, there were 44.2% of the total number of ASVs in N4RP and 62.6% of the total number of ASVs in N6RP for the corresponding years. There were 22.2% more bacterial ASVs in N6RP than in N4RP, showing an increase with increasing cultivation years. Whereas the number of bacterial ASVs in N6RS and N6BS showed the opposite trend, decreasing by 13.7% and 11.6% compared to N4RS and N4BS, respectively (Figure 1A). Concerning fungal ASVs, all ecological niches showed a decreasing trend with increasing years of cultivation. N6RP declined by 33.7% compared to N4RP, N6RS declined by 20.9% compared to N4RS, and N6BS showed the largest decline of 57.5% (Figure 1B).

Furthermore, NMDS analysis demonstrated differences in species composition among communities. The microbiomes of different cultivation years and niches were significantly separated on the NMDS1 and NMDS2 axes (Figures 1C,D). PERMANOVA analysis revealed that cultivation years significantly affected microbiomes at different soil distances. These differences were more pronounced for fungi than bacteria. N6 exhibited a greater impact on microbial taxa in different ecological niches of the root-soil than N4 (bacteria *R*^2^ = 0.5980, fungi *R*^2^ = 0.7703). The effect of cultivation years on the RS microbiome was relatively small (bacteria *R*^2^ = 0.3398, fungi *R*^2^ = 0.4119), but more on the BS microbiome (bacteria *R*^2^ = 0.5254, fungi *R*^2^ = 0.7474, Table 2).

**Table 2:** Results of PERMANOVA analysis.

Sample grouping	Short-term cultivated ecological niches N4 (RP vs. RS vs. BS)	Long-term cultivation ecological niches N6 (RP vs. RS vs. BS)	Rhizoplane (N4RP vs. N6RP)	Rhizosphere (N4RS vs. N6RS)	Bulk soil (N4BS vs. N6BS)
Df	2	2	1	1	1

Microbiota	Bacteria	Fungi	Bacteria	Fungi	Bacteria	Fungi	Bacteria	Fungi	Bacteria	Fungi
*R*^2^	0.5778	0.5095	0.5980	0.7703	0.4277	0.5562	0.3398	0.4119	0.5254	0.7474
Pr (>*F*)	0.0010	0.0010	0.0010	0.0010	0.0130	0.0110	0.0010	0.0030	0.0040	0.0100

Using Bray–Curtis distance measures, the *R*^2^ values indicate the proportion of variance explained by different subgroups in the sample set. *p* < 0.05 denotes a high level of significance.

### 3.3 Changes in the diversity of soil microorganisms

**Figure 2:**
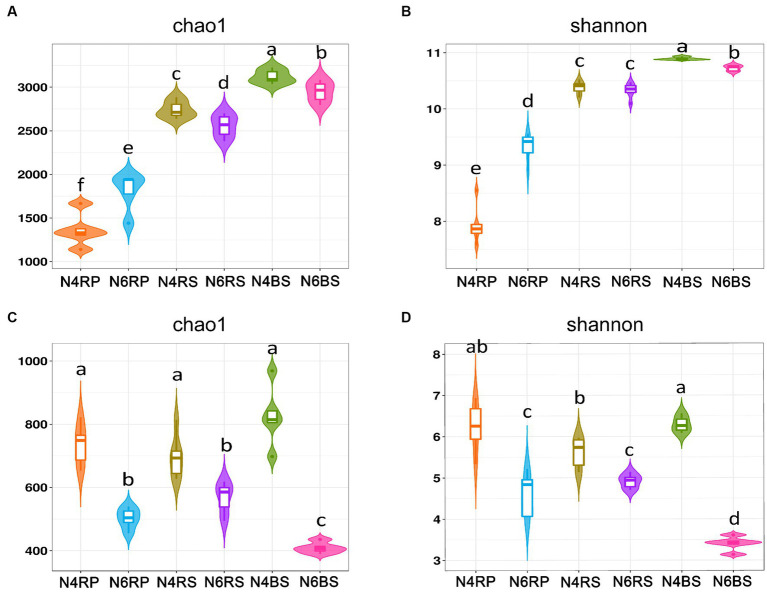
Alpha-diversity of microorganisms in ginseng at different ecological niches and years of cultivation. Chao 1 for bacteria **(A)**, Shannon for bacteria **(B)**, Chao 1 for fungi **(C)**, and Shannon for fungi **(D)**. Data are presented as the mean ± standard error (*p* < 0.05, according to the Kruskal–Wallis test).

In this study, we used the Chao1 and Shannon indices for assessing species richness and diversity, respectively. Regarding bacterial α-diversity, each ecotope showed different trends at various cultivation years. The bacterial α-diversity for each ecological niche exhibited a decreasing trend with increasing cultivation years in both the RS and BS. However, for N6RP, it was significantly larger than N4RP (N4RS > N6RS, N4BS > N6BS, Figures 2A,B). In a detailed comparison of bacterial α-diversity at different ecological niches, both Chao1 and Shannon indices showed a significant decreasing trend from “T-J-B” in N4 and N6 (Figures 2A,B). Regarding fungal α-diversity, both the Chao1 and Shannon indices showed significant decreasing trends in all ecological niches in N6 compared with N4. Especially in N6BS, which was significantly lower than the other niches and showed a cliff-like decline (Figures 2C,D).

### 3.4 Composition of the soil microbial community at the phylum and genus levels

**Figure 3:**
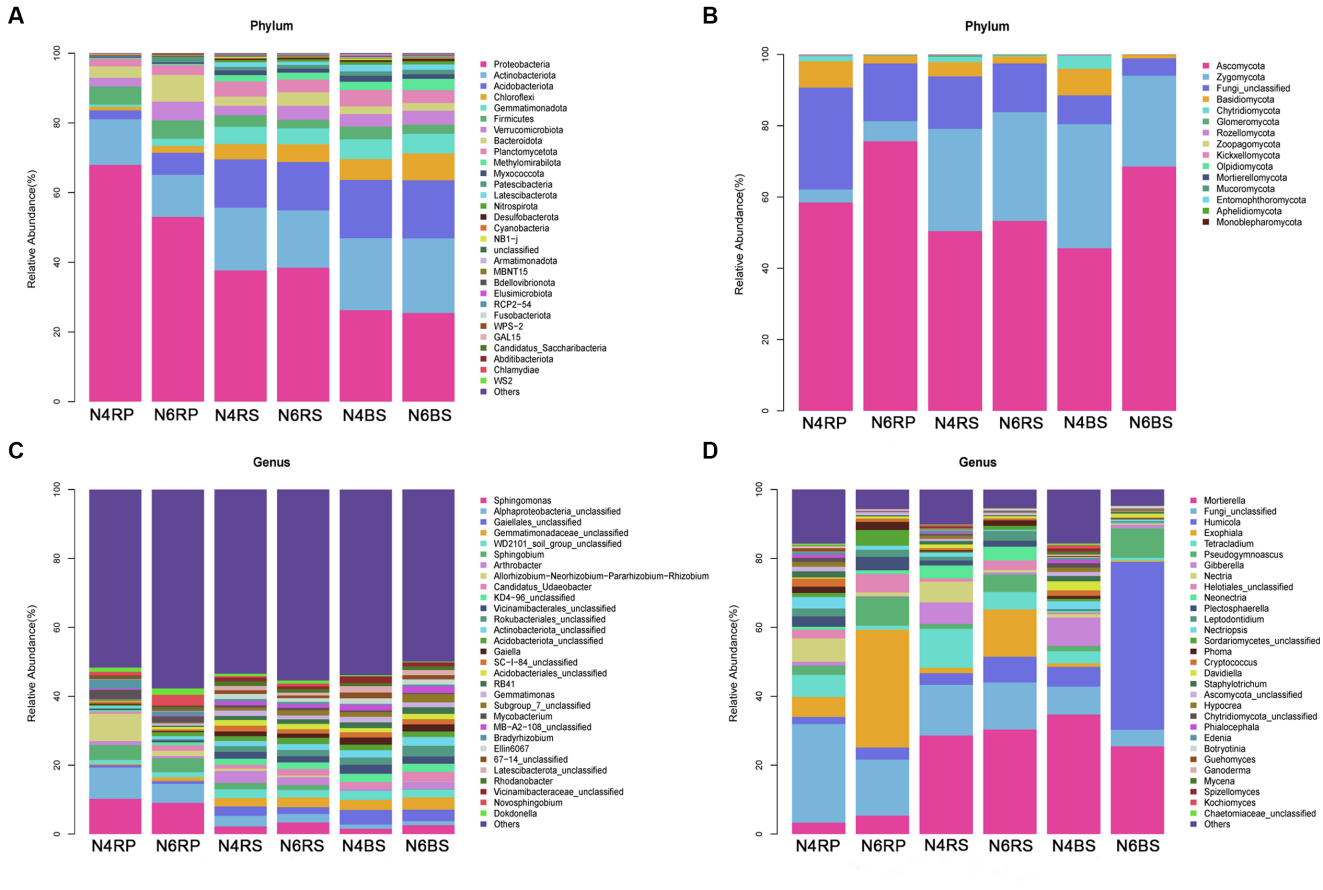
Species abundance stacked bar chart (TOP30). Average relative abundance change of bacterial phylum **(A)** and fungal class **(B)**, bacterial genus **(C)**, and fungal genus **(D)**.

Based on the obtained ASV feature sequences, we selected the top 30 species in terms of abundance for detailed classification at the phylum and genus levels. Among them, microorganisms with relative abundance greater than 1% were considered dominant. There were eight dominant bacterial phyla: Proteobacteria (relative abundance 25.47%–67.99%), Actinobacteriota (12.07%–21.42%), Acidobacteriota (2.56%–16.71%), Chloroflexi (1.03%–7.73%), Firmicutes (2.45%–5.27%), Verrucomicrobiota (2.44%–5.35%), Bacteroidota (2.20%–7.64%), and Planctomycetota (2.23%–4.34%). Proteobacteria was particularly dominant in the RP, decreasing with increasing cultivation years (N4RP: 67.99%, N6RP: 53.08%). This trend was also observed from “rhizoplane-rhizosphere-bulk soil” in both N4 and N6. Actinobacteriota also decreased with increasing cultivation years (N4RP: 13.07%, N6RP: 12.07%). However, all other dominant bacterial phyla, except Proteobacteria and Actinobacteria, showed an increasing trend in the RP with increasing cultivation years (Figure 3A).

Likewise, three main dominant fungal phyla were identified: Ascomycota (45.66%–75.68%), Zygomycota (3.65%–34.81%), and Fungi_unclassified (4.84%–28.59%). Ascomycota dominated all ecological niches, along with Zoopagomycota, increasing across all niches with increasing cultivation years. Zygomycota also increased in the RP and RS with more cultivation years. Most other fungal phyla showed a decreasing trend in all ecological niches with increasing cultivation years (Figure 3B).

At the genus level, *Sphingomonas* bacteria exhibited a higher abundance in N4RP (7.96%, Figure 3C) and showed a decreasing trend in N6RP (1.58%, Figure 3C). Regarding fungi, *Pseudogymnoascus* increased with more cultivation years, showing an increase of 2.41-fold, 2.55-fold, and 4.32-fold in the RP, RS, and BS, respectively (N4RP: 2.73%, N6RP: 8.43%, N4RS: 1.44%, N6RS: 5.11%, N4BS: 1.61%, N6BS: 8.56%). *Tetracladium* showed a decreasing trend with more cultivation years, dipping by 81.28%, 55.25%, and 77.39% in N6RP, N6RS, and N6BS, respectively (N4RP: 6.41%, N6RP: 1.20%, N4RS: 11.31%, N6RS: 5.06%, N4BS: 3.45%, N6BS: 0.78%). Also, *Exophiala* in N4RP and N4RS increased with more cultivation years (N4RP: 5.81%, N6RP: 34.18%, N4RS: 1.58%, N6RS: 13.71%). In contrast, *Humicolala* dramatically increased in BS (N4BS: 5.72%, N6BS: 48.72%, Figure 3D).

### 3.5 Differences in soil microbial community

**Figure 4:**
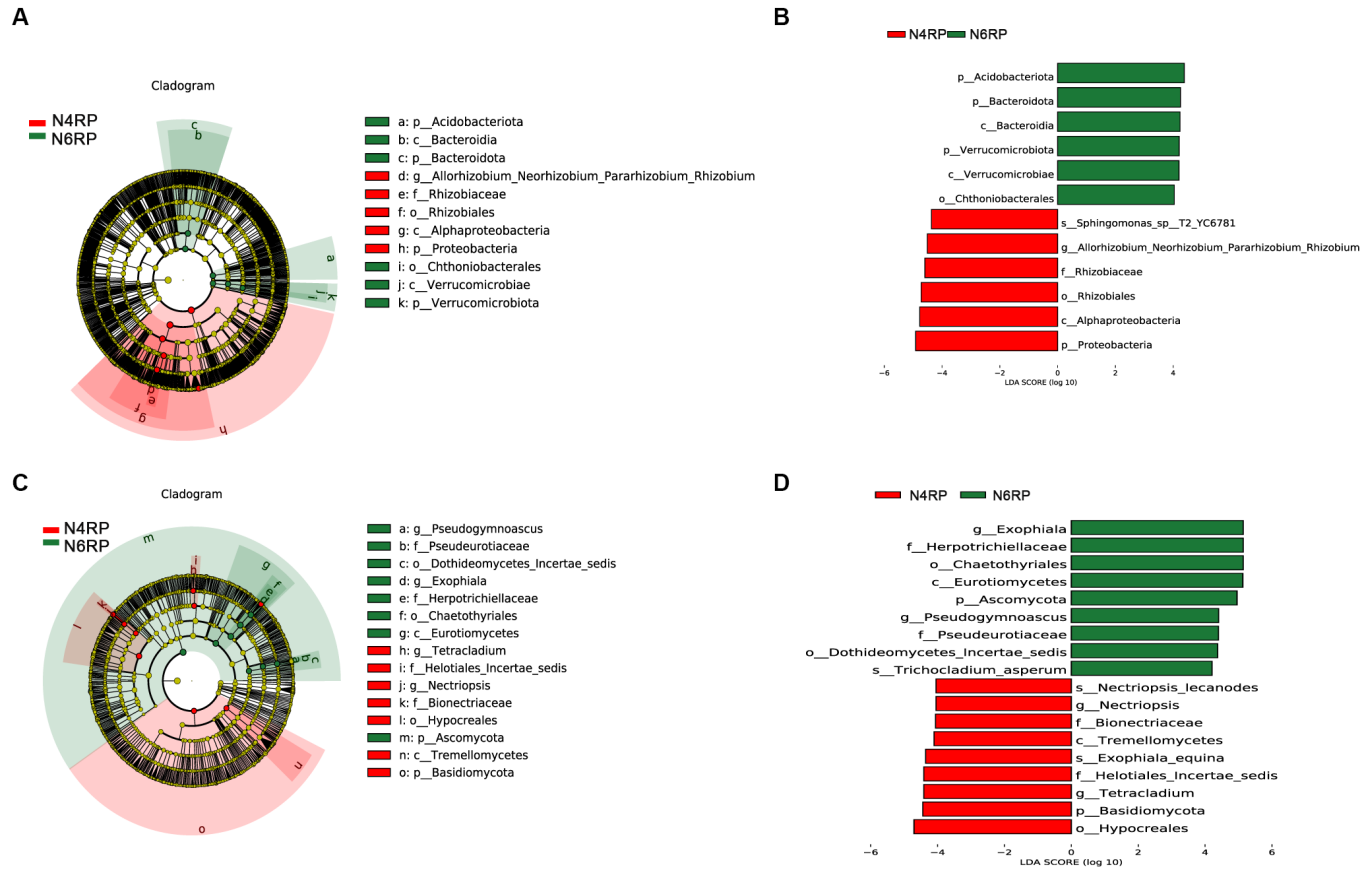
LEfSe analysis of rhizoplane soil samples. Arcograms of taxonomic evolutionary branches of two groups of significantly different bacteria **(A)** and fungi **(C)**, with different circle layers radiating from inside to outside representing the six taxonomic levels of the phylum. Phylum orders families and genera respectively, and each node representing a species classification at that level, with the greater the abundance of the species the greater the color range. The node color is yellow indicates that the species has no significant difference in the comparison group, the node color is red indicates that the species has significant difference species (i.e., biomarker) in the 4 years-old ginseng samples, and the node color is green indicates that the species has significant difference species (i.e., biomarker) in the 6 years-old ginseng samples, and the gates of the significant differences are directly labeled in the figure, and the other levels of the differences in species nodes were identified with letters to identify the specific species represented (Figures 5, 6, 8 and so on). Distribution of linear discriminant analysis (LDA) effect sizes for bacteria **(B)** and fungi **(D)** at seven taxonomic levels from phylum to species between the two groups, with the x-axis indicating LDA scores (log10) and the *y*-axis indicating significantly different bacterial and fungal (LDA >4.0).

**Figure 5:**
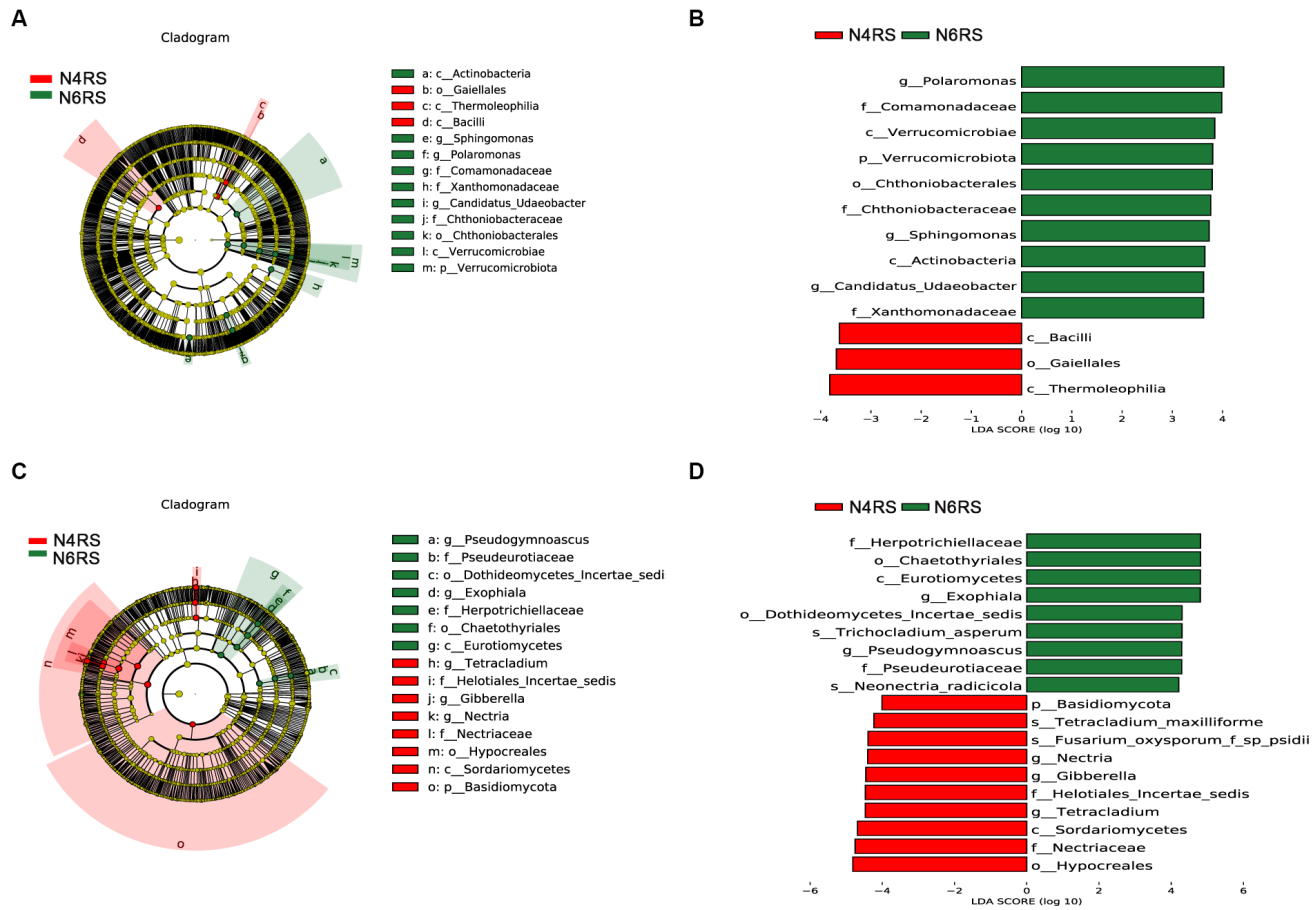
LEfSe analysis of rhizosphere soil samples. Arc plots of taxonomic evolutionary branches of bacteria **(A)** and fungi **(C)** with significant differences between the two groups. Distribution of linear discriminant analysis (LDA) effect sizes for bacteria **(B)** and fungi **(D)** between the two groups (bacterial LDA >3.5, fungal LDA >4.0).

**Figure 6:**
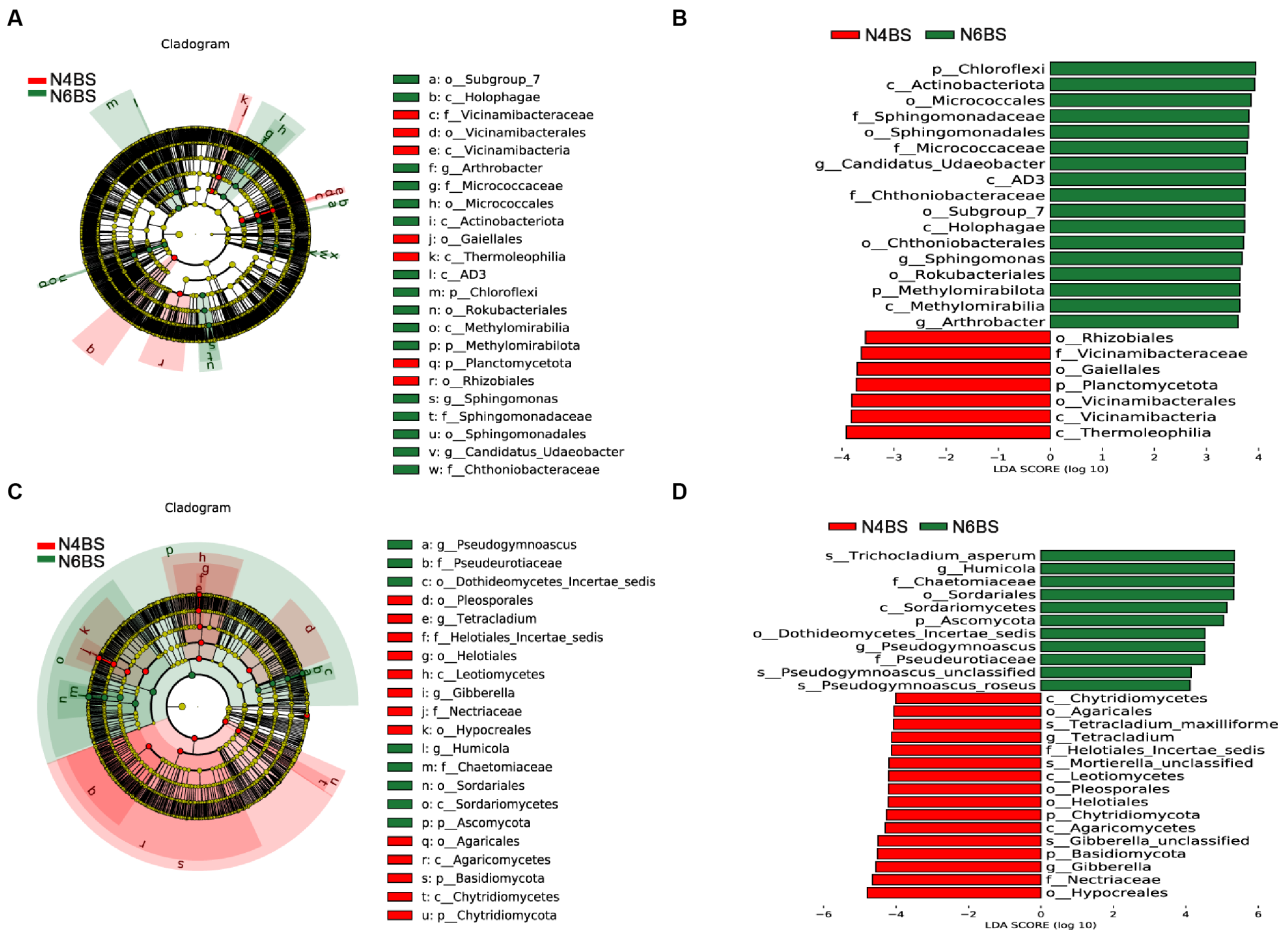
LEfSe analysis of bulk soil samples. Arc plots of taxonomic evolutionary branches of bacteria **(A)** and fungi **(C)** with significant differences between the two groups. Distribution of linear discriminant analysis (LDA) effect sizes for bacteria **(B)** and fungi **(D)** between the two groups (bacterial LDA >3.5, fungal LDA >4.0).

We conducted a thorough analysis of the significant enrichment of indicator species in each ecological niche affected by increasing cultivation years (Figures 4–6). We found that N4RP was significantly enriched for 5 bacteria and 7 fungi (Figure 4), including the bacteria: *Alphaproteobacteria*, *Rhizobiaceae*, *Allorhizobium_Neorhizobium_Pararhizobium_Rhizobium*, etc. (Figures 4A,B), and the fungi: *Nectriopsis*, *Tetracladium*, *Bionectriaceae*, etc. (Figures 4C,D). N6RP was significantly enriched for six bacteria and eight fungi (Figure 4), including the bacteria: *Bacteroidia*, *Chthoniobacterales*, etc. (Figures 4A,B), and the fungus: *Exophiala*, *Pseudogymnoascus*, *Herpotrichiellaceae*, etc. (Figures 4C,D). N4RS was significantly enriched for three bacteria and eight fungi (Figure 5), including bacteria: *Bacilli*, *Thermoleophilia*, *Gaiellales* (Figures 5A,B), and fungi: *Tetracladium*. N6RS was significantly enriched for 10 bacteria and 7 fungi (Figure 5), including bacteria: *Sphingomonas*, *Candidatus_Udaeobacter*, etc. (Figures 5A,B), and fungi: *Exophiala*, *Pseudogymnoascus*, etc. (Figures 5C,D). N4BS was significantly enriched for 7 bacteria and 13 fungi (Figure 6), including fungi: *Tetracladium*, *Gibberella*, etc. (Figures 6C,D). N6BS was significantly enriched for 17 bacteria and 13 fungi (Figure 6), including bacteria: *Sphingomonas*, *Candidatus_Udaeobacter*, *Arthrobacter*, and fungi: *Humicola*, *Pseudogymnoascus*, etc. (Figures 6C,D).

### 3.6 Prediction analysis of functional genes of microbial metabolic pathways

**Figure 7:**
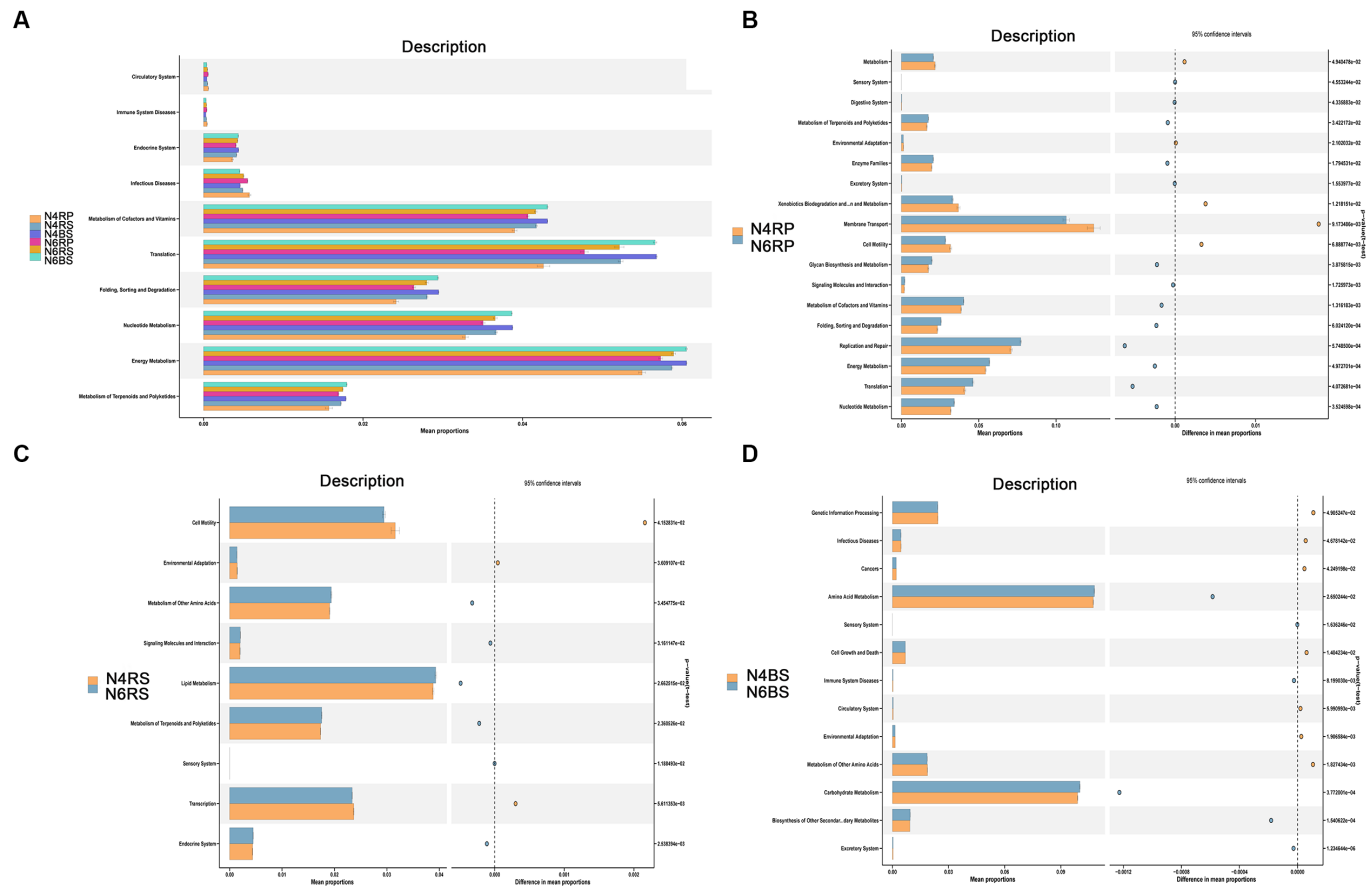
Potential functional roles of bacterial taxa across different ecological niches in the root system. **(A)** Using PICRUSt2, we predicted bacterial community differences between the rhizoplane (RP), the rhizosphere soil (RS), and the bulk soil (BS), in different ecological niches and years of cultivation in the root system. **(B–D)** Rhizoplane (RP), the rhizosphere soil (RS), and the bulk soil (BS) in different years of cultivation at KEGG level 2. Differences with *p* < 0.05 are visualized using stamp analysis, and significance was ascertained using Welch’s *t*-test.

The PICRUSt2 functional prediction results indicated high similarity in functional taxa and distributions between N4 and N6 ecotopes across different cultivation years (Figure 7A). A comparison of each ecotope revealed that functional classes in N6RP and N6RS were generally significantly higher than those in N4RP and N4RS (Figure 7). For instance, N6RP was significantly enriched in the metabolism of terpenoids and polyketides, signaling molecules and interaction, glycan biosynthesis and metabolism, energy metabolism, and translation, while N4RP was enriched in environmental adaptation, xenobiotics biodegradation and metabolism, membrane transport, and cell motility functions (Figure 7B).

The RS functions showed the least variation with increasing cultivation years, indicating greater stability in bacterial functions compared to other niches. N6RS exhibited significant enrichment in lipid metabolism, signaling molecules and interaction, and terpenoids and polyketides metabolism with increasing cultivation years. Conversely, N4RS displayed significant enrichment in cell motility, environmental adaptation, and transcription functions with fewer cultivation years (Figure 7C). Carbohydrate metabolism was enriched in N6BS, while amino acid metabolism and other functions were significantly enriched in N6BS (Figure 7D).

### 3.7 Correlation analysis of soil properties and enzyme activities with microorganisms

**Figure 8:**
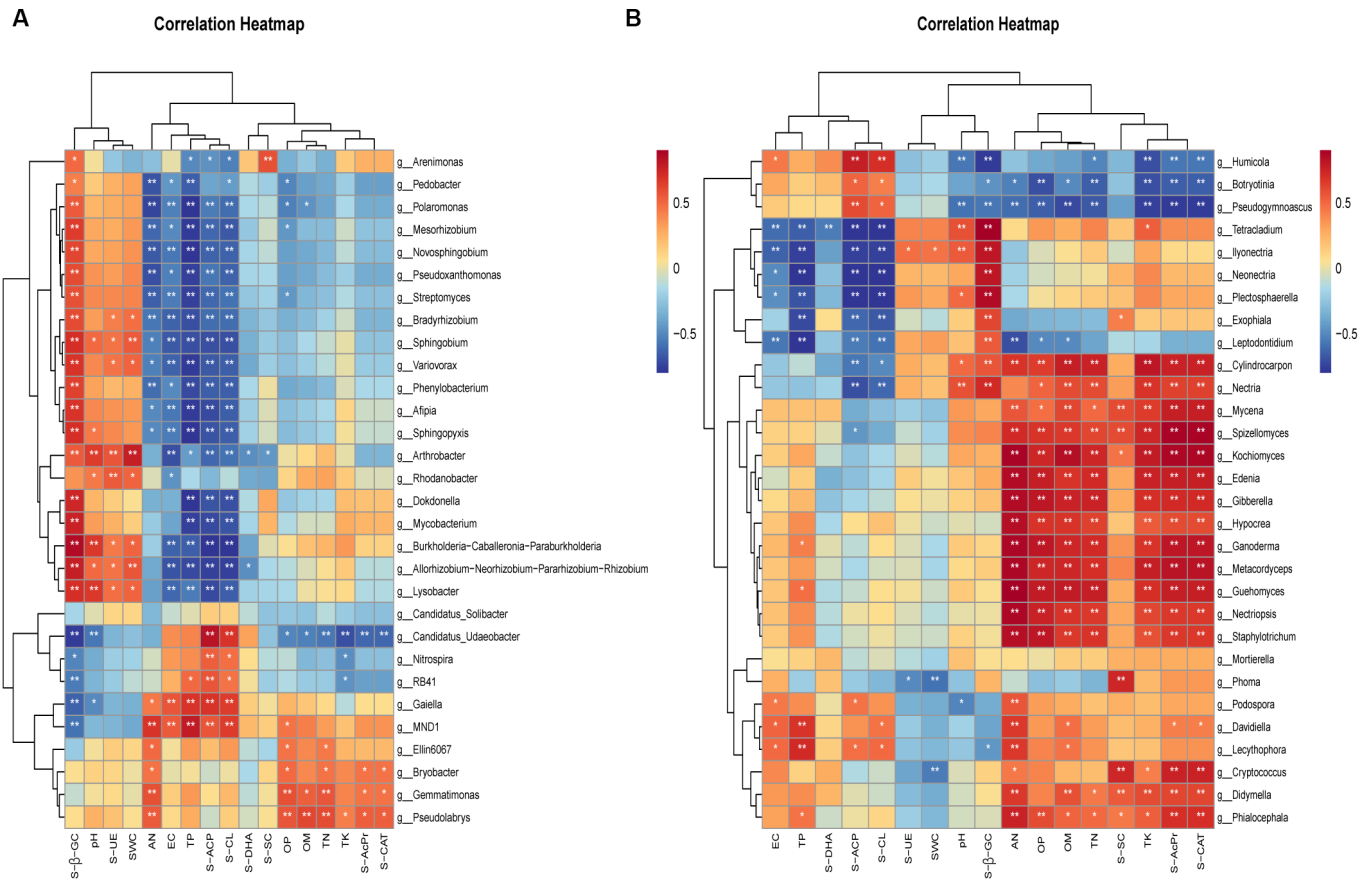
Spearman correlation coefficients between bacterial **(A)** and fungal **(B)** communities and environmental variables. The figure’s rows correspond to the top 30 species by abundance, and columns represent various environmental indicators. Significance levels are indicated as follows: ^*^p < 0.05 and ^**^p < 0.01. Cells colored in red and blue signify positive and negative correlations, respectively. Soil water content (SWC), pH, electrical conductance (EC), soil organic matter (SOM), total nitrogen (TN), total phosphorus (TP), total potassium (TK), available nitrogen (AN), and available phosphorus (OP), soil cellulase (S-CL), soil β-glucosidase (S-β-GC), acid protease (S-AcPr), soil urease (S-UE), soil acid phosphatase (S-ACP), soil sucrase (S-SC), soil catalase (S-CAT), soil dehydrogenase (S-DHA).

To gain insight into the relationship between soil microorganisms and soil physicochemical indicators and enzyme activities, we selected the top 30 bacterial and fungal genera. We then performed correlation clustering labeling heat map analysis. The results showed that the abundance and distribution of bacteria were mainly affected by pH, SWC, TP, AP, TN, AN, and SOM. Further analysis revealed significant positive correlations between bacteria and S-β-GC, S-UE, S-ACP, and S-CL (Figure 8A). Compared to bacteria, fungi were more susceptible to soil physicochemical properties such as pH, TN, AN, TK, TP, AP, and SOM. They also exhibited significant positive correlations with S-β-GC, S-SC, S-ACP, and S-CAT (Figure 8B).

## 4 Discussion

The microbiome of plants is a very complex and dynamic system. Studies have shown that microbial taxa are mainly influenced by soil environment, host genotype, ecological niche, and cultivation years (Wang et al., 2021; Xiong et al., 2021a,b). Gao et al. (2021) demonstrated that ecological niches have a greater influence on the soil microbiome than cultivation locations. Our study adds new knowledge by exploring the heterogeneity of microbial taxa in ginseng soils at different ecological niches. We found that microbial taxa were affected by cultivation years in different ecological niches, including RP, RS, and BS (Figures 1C,D and Table 2), and the differences increased with cultivation years (Table 2).

We revealed that the number of ASVs in the RP was the lowest among all ecological niches in ginseng RS soils of different cultivation years (Figure 1A). The bacterial α-diversity indices (Chao1 and Shannon’s index) showed a significant decreasing trend from “T-J-B” across different cultivation years (Figures 2A,B). This confirms the existence of a layer-by-layer selection pattern among ecological niches in the ginseng RS (Edwards et al., 2015; Huang et al., 2022). Different ecological niches have different screening strengths for microbial communities. This may be related to factors such as soil conditions, plant root secretions, and other factors (Jiang et al., 2021; Huang et al., 2022). Plants regulate their RS microbial environments through inter-root secretions (Hu et al., 2018). They screen and recruit specific microbial taxa in different ecological niches (Sun et al., 2021; Huang et al., 2022) for different roles (Huang et al., 2019, 2022; Shakir et al., 2021; Xiong et al., 2021b).

Under optimal conditions, RS microorganisms construct a protective barrier for the plant’s roots to defend against pathogens through interspecific competition and the production of antimicrobial substances (Li et al., 2019; Hu et al., 2020; Gao et al., 2021). We found that the RP microbial community exhibited the greatest stability with limited sensitivity to the number of cultivation years compared to the RS and BS (Table 2). This finding highlights the central role and inherent resilience of rhizospheric microbial communities in agroecosystems. We believe that the protective barrier for roots mainly exists in the RS soil within 2 nm from the roots. Ginseng roots of different cultivation years maintained a stable balance of microbial taxa in the RS soil ecotope. This was done through regulation, such as RP secretion. The diversity of microbial communities underpins ecosystem equilibrium (Hector and Bagchi, 2007; Isbell et al., 2011; Delgado-Baquerizo et al., 2016). In contrast, the RP and BS microbial communities, particularly in the BS, showed the most variability and least stability under varying cultivation years (Table 2). This was primarily evident in the marked “cliff” decline of fungi (Figure 2). This validates our previous studies showing that the species diversity of soil microorganisms gradually decreases with increasing cultivation years of ginseng planting (Xiao, 2015; Xiao et al., 2016).

Eutrophic bacteria mainly proliferate in nutrient-rich environments. Plant roots secrete many substances that are easily assimilated by microorganisms, such as sugars and amino acids. These substances provide a rich carbon source for RP microorganisms (Zhang et al., 2017; Yang et al., 2023). In the RP soil, the eutrophic bacterium *Ascomycetes* (Proteobacteria) was the predominant species. However, its relative abundance decreased from 67.99% to 53.08% with the increase in cultivation years (Figure 3A). This indicates that the effective carbon source or other nutrients in the RP soil may have decreased with increasing cultivation years. Oligotrophic bacteria reproduce slowly and predominantly inhabit environments with fewer nutrients (Jiang et al., 2021). We showed that the oligotrophic bacteria *Acidobacteriota*, *Chloroflexi*, *Gemmatimonadota*, and *Planctomycetota* showed a gradual increase from the “rhizoplane—rhizosphere—bulk soil” in different growth years (Figure 3A). This suggests significant changes in soil physicochemical properties, as total nitrogen, quick-acting nitrogen, and quick-acting phosphorus became significantly lower in the RS with higher cultivation years (Table 1). We, therefore, hypothesized that nutrient utilization decreases from the “rhizoplane—rhizosphere—bulk soil” irrespective of cultivation years. Nutrient utilization in the higher cultivation years’ RS led to a notable decrease in nutrient content. The relative abundance of the nutrient-rich fungus *Ascomycota*, known to promote ginseng’s resistance to pathogens (Wang et al., 2021), increased in each ecotope with years of cultivation. This suggests that nutrient content in the RP ecotopes could increase over the years, potentially enhancing disease resistance.

The bacterium *Sphingomonas*, relatively abundant in RP soils (Figure 3B), significantly enriches the ginseng RS and BS in high vintages (Figures 5, 6). It promotes plant yield by degrading toxic soil compounds, defending against pathogenic bacteria, and regulating plant metabolism (Liu et al., 2023). Additionally, it contributes to nitrogen fixation, phosphate solubilization, and plant growth hormone production (Asaf et al., 2020; Kim et al., 2020; Shi Y. et al., 2022). It also improves plant growth under specific environmental stresses such as drought, high salinity, and heavy metal stress (Asaf et al., 2020). Prolonged ginseng cultivation leads to harmful substance accumulation in the soil, thus increasing these beneficial bacteria. The beneficial fungus *Tetracladium* had a high relative abundance of 11.31% in the RS soil with shorter years of cultivation. It was significantly enriched in all soil ecological niches with lower cultivation years (Figure 3D). Studies indicate a mutually exclusive relationship between *Tetracladium* and root pathogens, potentially inhibiting certain pathogens (Hilton et al., 2021). Furthermore, *Tetracladium* is positively associated with crop yield, benefiting host health and growth (Lazar et al., 2022). In ginseng cultivated for long periods, the fungus *Exophiala* becomes significantly enriched in the RP and RS (Figures 5C,D, 6C,D). Its relative abundance in the RS soil reaches as high as 34.18% (Figure 3D). Similarly, *Humicola* was significantly enriched in ginseng BS in later years (Figures 7C,D). Its relative abundance went up to 48.72% (Figure 3D). These fungi are known as pathogen-inhibiting fungi. This suggests that succession disorder in plant ginseng has a strong “cry for help” strategy under normal growth. Additionally, each ecological niche significantly recruits a high proportion of pathogen-suppressor fungi to adapt to the environment. However, the nature of how the root secretion “cry for help” operates needs to be further elucidated. This requires macrogenomics and metabolomics studies to clarify the mode of “rhizosphere talk.”

We previously showed that some potentially pathogenic fungi, such as *Cryptococcus* spp. and *Fusarium* spp., were present only in the soil of cultivated ginseng (Xiao et al., 2016). We found that ginseng soils with many cultivation years significantly accumulate the phenolic acid-producing fungus *Pseudogymnoascus* in all ecological sites (Figures 5–7). In particular, in BS, the accumulation increases by a factor of 4.32 (Figure 3D). We previously showed that phenolic acid accumulation in cultivated ginseng soil leads to chemosensitization (Yang et al., 2017). Phenolic acids differentially regulate RS soil microbial communities. They promote pathogenic bacteria proliferation while attenuating beneficial bacteria (Wu et al., 2017). Moreover, phenolic acids enhance the mycelial growth of root rot pathogens and slow down alfalfa growth (Wu et al., 2017). The fungus *Pseudogymnoascus* produces phenolic acid compounds like vanillic acid (Guo et al., 2019). These compounds promote pathogenic bacteria growth, disrupting the microecological balance and affecting ginseng cultivation succession. We hypothesize that ginseng soil acidification in high vintage years is not solely due to plant root-secreted phenolic acid compounds. It also relates to microorganisms producing these compounds, which are significantly enriched in various ecological niches. Soil physicochemical properties are the most important environmental factors affecting microbial and functional gene composition (Jiang et al., 2021; Hagh-Doust et al., 2023). We found that fungal communities were more sensitive to ginseng cultivation years and soil physicochemical properties relative to bacteria (Table 1 and Figure 7). This is consistent with several previous studies (Dini-Andreote and Raaijmakers, 2018; Zhong et al., 2018; Wang et al., 2021). Bacterial communities exhibit more stable characteristics due to their rapid reproduction ability and strong resistance to environmental disturbances (Wang et al., 2021). In contrast, fungi have relatively weak resistance, signifying the importance of regulating fungal homeostasis. Overall, soil physicochemical properties such as pH, TN, AN, TP, OP, and SOM significantly influenced ginseng soil bacteria and fungi (Figure 7), and the effect was more pronounced on fungi. Xiao (2015) and Philippot et al. (2023) demonstrated a significant decrease in ginseng RS soil SOM, AN, etc., with the increasing years of cultivation, which is consistent with our results (Table 1). TN, AN, OP, etc., were significantly lower than the other groups in the RS soil in the higher years of cultivation. This indicated a large differential effect of the cultivation year on the physicochemical properties of ginseng soils at different ecological niches.

Numerous studies have demonstrated that plants have specific microbial communities and functions when grown in different soils (Huang et al., 2019; Li et al., 2019). We used a 3 years-old ginseng seedling transplantation and hypothesized that in the first year of cultivation, the microbial taxa present on the RP and in the RS were mainly enriched with functional genes. These genes were linked to cell motility and environmental adaptation (Figure 8). They help adapt to the environment in the plants’ new environments. The microbial taxa were sufficiently shaped to their specific RS microbiota in these new settings (Lu et al., 2018). As ginseng cultivation years increased, most functional genes involved in the metabolism of terpenoids and polyketides, signaling molecules, and interaction became significantly enriched (Figure 8). Studies have shown that plants can attract beneficial microorganisms at a distance by actively releasing nonvolatile root secretions or mixtures of organic compounds (Schulz-Bohm et al., 2018; Gao et al., 2021). Huang et al. (2019) demonstrated that 52% of Arabidopsis root-specific bacteria are related to triterpene synthesis. Triterpenoids directly and selectively regulate RS bacterial growth and participate in the co-evolution of the plant and RS microbiomes. We think that terpenoids and polyketides play an important role in ginseng-mediated regulation of microbial taxa in the RP and RS ecological niches. In addition, we found that functional genes in the RS were most stable across cultivation years compared to other ecotopes (Figure 8). This suggests that the RS ecotope 0–2 nm away from the root is of high importance. It is crucial to investigate how host cultivation years regulate the stability of the RS microbial taxa. Macro-genomics and metabolomics can be further utilized to investigate rhizospheric interactions that may help understand the precise regulation of plant microbiomes.

## 5 Conclusion

We showed that during normal growth, the succession disorder plant ginseng exhibits a “cry for help” strategy. To adapt to the environment, each ecological niche recruits a significant proportion of pathogen-suppressing fungi. With an increasing number of ginseng cultivation years, the number of beneficial bacteria in the RP soil decreases, and that of pathogen-inhibiting fungi increases in the RS and BS. There is a cliff-like decline in fungal α-diversity, alongside soil acidification. The microecological balance, dominated by BS ecotopes, becomes disrupted. Meanwhile, a significant increase occurs in the phenolic acid-producing fungus *Pseudogymnoascus* in the root soil. This led to soil acidification, altering microbial groups. These microbial changes, in turn, worsened soil acidification and reduced the dynamics of the soil microbial environment. This cyclic effect ultimately causes succession disorder. Therefore, farmers must pay attention to the microbial environmental changes in each ecological niche of the soil during the long-term cultivation of ginseng. We must take measures to maintain and restore the soil’s ecological balance, especially in the BS ecological niche. Improving soil physicochemical properties can regulate change in microbial communities, and keeping soil physicochemical properties can influence shifts in microbial communities. Maintaining soil ecological balance is crucial for preventing succession disorder. Nevertheless, here, we only conducted 16S rDNA and ITS studies on each ecological niche of the ginseng soil microbiome. In the future, we plan to conduct metagenomics and macro metabolomic studies to better understand microbial resources for healthy and sustainable ginseng cultivation.

## Data availability statement

The datasets presented in this study can be found in online repositories. The names of the repository/repositories and accession number(s) can be found at: https://www.ncbi.nlm.nih.gov/, PRJNA1004743 https://www.ncbi.nlm.nih.gov/, PRJNA1004746.

## Author contributions

ZS: Writing – original draft, Writing – review & editing. MY: Writing – original draft. KL: Writing – original draft. LiY: Writing – review & editing. LimY: Writing – review & editing.
