# Supplementary material for: Influence of cultivation duration on microbial taxa aggregation in Panax ginseng soils across ecological niches
Source: Front Microbiol. 2024 Jan 10;14:1284191. doi: 10.3389/fmicb.2023.1284191 (PMC10813202; doi:10.3389/fmicb.2023.1284191)
Supplement: Supplementary file 1 [file Table_1.docx]

Influence of cultivation duration on microbial taxa aggregation in *Panax ginseng* soils across ecological niches

Zhenting Shi, Meiling Yang，Kexin Li， Li Yang^*^, Limin Yang^*^

Cultivation base of State Key Laboratory for Ecological Restoration and Ecosystem Management，College of Traditional Chinese Medicine，Jilin Agricultural University, Changchun，China

*** Correspondence:**

Limin Yang

[ylmh777@126.com](mailto:ylmh777@126.com)

Li Yang

yangliff@126.com

**Table S1 Weight of ginseng at different years of cultivation (P=0.05)**

| groups | number | fresh weight (g / branch) |
| --- | --- | --- |
| 4 years old ginseng | N4-1 | 10.71±4.123b |
|  | N4-2 | 11.26±3.984b |
|  | N4-3 | 14.86±5.651b |
|  | N4-4 | 13.18±4.146b |
|  | N4-5 | 13.88±4.788b |
|  | N4-6 | 11.25±6.12b |
| 6 years old ginseng | N6-1 | 36.1±21.8a |
|  | N6-2 | 25.64±18.05ab |
|  | N6-3 | 28.2±11.7a |
|  | N6-4 | 31.29±16.96a |
|  | N6-5 | 25.91±17.72ab |
|  | N6-6 | 28.2±17.33ab |
